# Etiological heterogeneity and clinical variability in newborns with esophageal atresia

**DOI:** 10.1186/s13052-018-0445-5

**Published:** 2018-01-26

**Authors:** Ettore Piro, Ingrid Anne Mandy Schierz, Mario Giuffrè, Giovanni Cuffaro, Simona La Placa, Vincenzo Antona, Federico Matina, Giuseppe Puccio, Marcello Cimador, Giovanni Corsello

**Affiliations:** 1 Neonatal Intensive Care Unit, A.O.U.P. “P. Giaccone”, Department of Sciences for Health Promotion and Mother and Child Care “G. D’Alessandro”, University of Palermo, Via Alfonso Giordano n. 3, Palermo, 90127 Italy; 2 Pediatric Surgical Unit. A.O.U.P. “P. Giaccone”, Department of Sciences for Health Promotion and Mother and Child Care “G. D’Alessandro”, University of Palermo, Via Alfonso Giordano n. 3, Palermo, 90127 Italy

**Keywords:** Retrospective study, Esophageal atresia, VACTERL association, Neonatal intensive care, Newborn

## Abstract

**Background:**

The aim of this study was to define different characteristics of infants with esophageal atresia and correlations with neonatal level of care, morbidity and mortality occurring during hospital stay.

**Methods:**

Charts of all newborns with esophageal atresia (EA) admitted to our University NICU between January 2003 and November 2016 were reviewed and subdivided in four groups related to different clinical presentations; EA as an isolated form (A), with a concomitant single malformation (B), as VACTERL association (C), and in the context of a syndrome or an entity of multiple congenital anomalies (D).

**Results:**

We recruited 67 infants with EA (with or without tracheoesophageal fistula), distributed in groups as follows: A 31.3%, B 16.4%, C 26.8% and D 25.3%. Type of atresia was not statistically different among different groups. Mortality was higher in groups C and D, especially if associated with congenital heart defects.

In survivors, we found different auxological evolution and prognostic profiles considering duration in days of invasive mechanical ventilation and total parenteral nutrition, as well as length of stay and corrected gestational age at discharge.

**Conclusions:**

In the context of genetic and syndromic entities, subjects with VACTERL association showed a lower mortality rate although a higher and more complex level of intensive care was noted in comparison to infants without VACTERL genetic and syndromic entities.

## Background

First documented in 1670 in a thoracophagus-conjoined female twin, esophageal atresia (EA) was considered a universally fatal anomaly. In the fifth decade of the twentieth century, in relation to failure of palliative measures, pediatric surgeons began to successfully treat these infants by extrapleural approach, tracheoesophageal fistula (TEF) ligation and single layered esophageal anastomosis [1]. Despite the refined surgical approach and highest quality improvements in perioperative neonatal care, these infants continue to be exposed to additional risks secondary to respiratory distress (RDS), total parenteral nutrition (TPN), medical devices and infections, with increased vulnerability in infants with low birth weight [2] and associated major malformations [3].

EA with and without TEF is part of a heterogenous spectrum of anatomic variants [4] that ranges from isolated EA to isolated TEF and are usually classified as A) EA without a TEF and associated with a long gap EA (LGEA); B) EA with a proximal TEF; C) EA with distal TEF, the most frequently encountered form of EA; D) EA with double TEF; E) TEF without EA; F) esophageal stenosis. The prevalence of EA in Europe is about 2.43 cases per 10,000 births (95% CI 2.30 to 2.57) [5], with a tendency to occur more often in boys, white race, first pregnancy, advanced mother age, siblings of patients with EA. in addition, about half of the cases are associated with other malformations and up to 10% of cases are found in specific chromosomal or single-gene disorders. Chromosomal abnormalities include trisomies (21, 13 and 18); 22q-, 13q-, 17q-, 16q24-microdeletion syndromes, Opitz syndrome. Single-gene mutations were responsible for Feingold syndrome, CHARGE syndrome (coloboma, heart defect, choanal atresia, growth retardation, genitourinary, and ear anomalies), AEG syndrome (anophthalmia and genital abnormalities), Pallister-Hall syndrome, Opitz G syndrome, Fanconi anemia-complex, Goldenhar syndrome, Beckwith-Wiedemann syndrome, Martines-Frias syndrome and VACTERL association [6]. We aimed to define different characteristics of infants with esophageal atresia and correlations with neonatal level of care, morbidity and mortality occurring during hospital stay.

## Methods

After approval from our Hospital Ethic Committee, we performed a retrospective review including all consecutive infants, inborn and outborn, affected by EA and hospitalized at the University Hospital NICU from January 2003 to November 2016. In the period considered, our NICU was the referral one for the unique first level neonatal surgery Unit in western Sicily and both neonatal intensive care and surgical staffs followed overall homogeneous treatment strategies in approaching newborns affected by EA.

Surgical repair and extrapleural ligation of fistula was performed as soon as possible after diagnosis through an extrapleural thoracotomy, and without preoperative tracheobronchoscopy. End-to-end anastomosis of esophageal segments was made in case of short gap EA (SGEA) and temporary gastrostomy with or without cervicostomy was the procedure of choice in case of LGEA.

After surgery, individualized analgosedation and mechanical ventilation has been continued and a trans-anastomotic feeding tube (TAFT) maintained in all infants with SGEA, to shorten TPN duration and decrease the risk of cholestasis.

We subdivided our patients into four groups; (A) EA as an isolated disease, (B) EA associated with a single concomitant malformation, (C) EA with at least two of vertebral defects, anorectal malformation, cardiovascular anomalies, renal malformation or anomalies of upper limbs, in the context of VACTERL association (OMIM #192350), and (D) EA in the context of a syndrome or an entity of multiple congenital anomalies with or without molecular genetic diagnosis.

We collected from medical records patient’s gestational age, multiple births, perinatal and anthropometric data, type of EA (“long gap” between the two ends of the esophagus was defined as a gap longer than 3 cm or greater than the height of 2 vertebral bodies) [7], day of surgery, complication, sepsis, comorbidity and mortality. In survivors until hospital discharge or transferred to Cardiac Surgery Unit, we considered duration of invasive mechanical ventilation (IMV), TPN, and duration of hospital stay.

We defined small for gestational age (SGA) all newborns with a birthweight less than 10th centile, according to Italian Neonatal Growth Charts (INES Charts) [8].

We analyzed the correlations among groups, clinical and prognostic parameters. Statistical analysis was performed by open source Statistical R Commander and *p*-value ≤0.05 was considered significant. Univariate analysis was conducted using for categorical variables chi-square and Fisher tests and for continuous variables t-tests, Wilcoxon and Kruskal-Wallis-tests, finally we performed a stepwise multivariate analysis [9].

## Results

We studied a sample of 67 newborns with EA, 36 males and 31 females, (54% versus 46%, *p = .*5431), with a prevalence of 3.1% of the total NICU admissions during the period considered.

The average birth weight was 2470 g (range, 500–4050 g) with an average gestational age at birth of 36.6-week (range, 25.1–41.4). Preterm infants were 29/67 (43%).

We subdivided the sample of infants with EA into four, well represented as number of patients, categories as described in the Materials and Method section: group A 21 patients (31.3%), B 11 patients (16.4%), C 18 patients (26.8%) and D 17 patients (25.3%).

We analyzed several variables considered at different levels as shown in Table 1.

**Table 1:** Distribution of several variables in the groups A, B, C and D

Variables (median), in total sample	Group A (*n* = 21)	B (*n* = 11)	C (*n* = 18)	D (*n* = 17)	*p*
Gestational age, weeks	39.4	37	35.8	36	**0.000075**
Mother’s age, years	32.5	32.7	30.6	33	0.81
Twinning, %	5	9	17	18	0.58
Absolute weight, g	2940	2640	2055	2000	**0.000033**
Weight centile	25	15	19.5	8	0.153
SGA, %	19	36	39	59	0.09
Head circumference centile	71	68	50	19	**0.0328**
Gross type					
A, %	9.5	9	5.5	6	0.64
C, %	90.5	82	89	94	0.30
D, %	0	9	5.5	0	0.21
Congenital heart defect, %	0	0	33	18	**0.005**
Apgar 1’	9	8	8	6	**0.045**
Apgar 5’	10	9	9	8	**0.028**
First surgery, day	2	2	2	2	0.1271
Gap distance (long gap), %	14	9	22	6	0.6
Infection, %	21	9	28	18	0.72
Mortality, %	10	0	28	53	**0.0028**
In survivors					
Time of intubation, days	6	5	9	7	**0.036**
Time of parenteral nutrition, days	8	10	13	9	**0.025**
Time of hospital stay, days	21	29	65	45	**0.00007**
Corrected gestational age at discharge, weeks	42.3	41.4	46.7	43.8	**0.025**

Significant differences are in bold

Among patient level variables we found in groups A and B a significantly higher median gestational age in comparison to group C and D (39.4–37–35.8-36 weeks, respectively; *p = .*000075), birth weight (2940–2640–2055-2000 g, respectively; *p = .*000033), head circumference centile (71–68–50-19; *p = .*0328), Apgar score at 1st and 5th minute (*p = .*045 and *p = .*0279, respectively), but not difference for multiple births (*p = .*15) and type of atresia (*p = .*79). SGA infants were prevailing in groups C and D, but not reaching statistical significance (*p = .*059).

Congenital heart defects (CHD) were more frequent in groups C and D versus B (60% versus 19% *p = .*0064), and severe CHD, including large ventricular septal defect and tetralogy of Fallot, were present only in group C and D (29%; *p = .*001) contributing to the higher mortality rate. Mortality variables analyzed by multiple logistic regression are reported in Table 2.

**Table 2:** Distribution, medians and Odds ratio of prognostic parameters

Variables (median)	Non-survivors (*n* = 16)	Survivors (*n* = 51)	*p*	Exponentiated coefficients/Odds-ratio
Gestational age, weeks	35.2	38	**0.001**	0.69
Weight, g	1810	2750	**0.0003**	1
Weight centile	15.5	21	0.39	1
Preterm rate, %	75	33	**0.0043**	5.8 (1.48, 28.6)
Twinning rate, %	25	8	0.08	3.82 (0.61, 23.8)
SGA, %	44	35	0.5418	1.41 (0.38, 5.14)
Head circumference centile	31.5	52	0.58	1
Major cardiopathy, %	31	8	**0.03**	5.17 (0.94, 30.79)
VACTERL-associated or syndromic EA, %	88	41	**0.0014**	9.7 (1.9, 96.66)
Apgar 1’	6	8	0.07	0.78
Apgar 5’	8	9	0.11	0.76
Surgical complication rate	6	16	0.33	0.36 (0.007, 3.11)
Non-surgical complication rate	12.5	18	1	0.67 (0.06, 3.8)
Waterstone classification class C, %	87.5	45	**0.0035**	8.28 (1.64, 82.67)
Montreal classification class II, %	87.5	45	**0.0035**	8.28 (1.64, 82.67)
Spitz classification class III, %	6	2	0.42	3.26 (0.03, 266.1)

Significant differences are in bold

Surgery level variables, including age in days at surgical intervention (median 2 days, range 1–5 days; *p = .*127), type of procedures, primary end to end anastomosis and TEF closure or temporary gastrostomy with or without cervicostomy (*p = .*71), showed not statistical significance among groups.

Following Gross classification, in the total sample, type C of EA was prevalent (90%), type A (7%) and D (3%) represented the remaining cases.

LGEA were 9/67 (13.4%) and equally distributed among the four groups (*p = .*396).

All but one infants with LGEA were treated by temporary gastrostomy. The only one with LGEA who underwent simple end to end anastomosis was a twin, preterm, with Beckwith Wiedemann syndrome.

Sepsis occurred in six infants (8.9%; *p = .*6615) and was successfully resolved in all cases by antibiotic treatment. Acute renal failure occurred in three infants; two with VACTERL association, and one with Charge syndrome, resulting lethal in two of them.

Early surgical complications (pneumothorax, chylothorax, anastomotic leakage, vocal cord paralysis) occurred in 15.6% of infants, without statistical difference (*p = .*33). One surviving preterm with VACTERL association suffered from a grade II intraventricular hemorrhage.

Presurgical death occurred in an extremely preterm with VACTERL association and an infant with trisomy 18.

Postsurgical death occurred in 14/65 infants (21.5%), affecting 6/36 males and 8/29 females (*p = .*3) and was higher in infants belonging to group C and D versus A and B (36.3% versus 6.25%; *p = .*008).

Considering in the global sample the worst prognostic class of the three classification systems (Waterston, Montreal and Spitz), we confirm a tight correlation with mortality except for the Spitz classification. These results can be partly related to the high mean birth weight present in the global sample.

Intensive level care variables were analyzed in survivors (51/67), length of IMV (*p = .*036) and length of TPN (*p = .*025) were statistically significant among groups. Particularly, patients in group C presented the longest duration of both IMV (9 days) and TPN (13 days). Anyway, TPN duration was not shortened by gastrostomy (7 versus 9 days in patients without gastrostomy; *p = .*42).

Length of hospital stay in survivors excluding two infants transferred for cardiac surgery, showed statistical significance for days (median 21, 29, 65, 45 days; *p = .*00007) as well as for corrected gestational age at hospital discharge (42.3, 41.4, 46.7, 43.8 weeks; *p = .*025). Interestingly, infants with VACTERL association showed the longest hospital stay related to the major global care complexity required by survivors in this group.

## Discussion

Management of infants with EA is still challenging despite advances in preterm birth prevention, neonatal intensive care management and surgical approaches have contributed to the improvement in survival rates. In recent papers, early and late morbidity as well as mortality have been considered as main outcomes and classifications aiming to define prognostic profiles of EA have been based on birth weight [2, 3, 10], pulmonary impairment and severity of congenital anomaly [11] and major cardiac anomaly [3, 10]. The management of infants with EA is characterized by a remarkable intensity level of neonatal assistance involving mainly individualized surgical approach, analgosedation, cardiorespiratory stabilization, and nutritional support. Considering the etiological heterogeneity of EA and the different anatomic and functional impairment, we aimed to investigate the various neonatal characteristics of infants with EA subdivided in four groups encompassing the main different presenting conditions that neonatologists are facing in relation to intensity level of provided neonatal assistance.

In the considered period, the surgical and neonatal intensive care as well as surgical staffs followed overall homogeneous treatment strategies in approaching newborns affected by EA, trying to intervene surgically as soon as possible once the diagnosis was made.

We divided our sample of 67 infants with EA in the aforementioned four groups. To our knowledge, the selected variables have been analyzed for the first time considering the different clinical profile of infants with EA. Therefore, our aim has been to identify similarities and differences among the four groups of infants regarding clinical characteristics and neonatal intensity level of care.

During the period considered the surgical team did not change the preoperative approach with no tracheobronchoscopy (TBS) performed before surgery, confirming a recent European survey demonstrating that only 43% of respondent consultants had routinely performed it before surgery [12].

### Auxological data

In our sample the high preterm birth rate (43%), universally considered a major risk factor for morbidity and mortality, was more frequent in groups C and D (mean gestational age 35 and 36 weeks respectively), versus A and B determining a concomitant statistically significant lower birth weight. Nevertheless, SGA condition, as already reported [13] was not prevalent in infants with a more complex malformation profile and was equally distributed among groups.

A higher malformation rate is reported in twins [14, 15], in our sample although twins with EA were more present in VACTERL and syndromic infants this finding was not statistically significant, probably related to the small sample.

The significantly lower head circumference centile found in group D can be considered as part of the more complex constitutional profile of such infants.

Despite of the high prevalence of CHD in our sample, we found severe CHD (tetralogy of Fallot and hemodynamically significant ventricular septal defect), only in group C and D, and never as isolated malformation concomitant to EA in group B, suggesting the opportunity of genetic counselling for the common embryological origin [16].

### Days at surgical intervention

Primary surgical repair of the esophagus and closure of the concomitant fistula is preferably performed in the first days to prevent pneumonia. In literature, mean interval time to surgery is ranging from 22 to 24 h [17] to 4 days [18], with longest interval to perform end to end anastomosis (mean 48.5 days) in case of syndromic LGEA or late surgical referral [19]. Our mean interval time to surgery of 2 days (range 1–5 days) was in line with early treatment strategy.

### Ventilatory support

Preoperative tracheal intubation is a negative prognostic factor [20] as shown in three infants in our sample. Two with VACTERL association both preterm and died after surgical repair and one at term, outborn, with isolated EA suffering from birth asphyxia and extubated before surgical repair.

In samples of infants with EA, considered as a whole, mean length in days of intubation is not always corresponding to days spent on IMV, because of maintenance of ET during CPAP [21]. Mean days of ventilatory support described is ranging from less than 4 days, with high frequency of extubation before 96 h [21] to 9.6 days (range 1–106 days) [18]. Preoperative intubation is considered an indicator of severe respiratory disease, and of other problem when >5 days [20]. Bagolan et al. [19] suggest, to prevent disruptive anastomotic force, to paralyze and assist with IMV all infants with EA for at least 6 days. In our sample analgosedation has been always associated with IMV and we registered in the four groups of patients a similar median length of IMV, ranging from 5 to 9 days, with a statistically significant longer duration in groups C and D (*p = .*036).

### TPN

Difficult weaning from post-surgical TPN (≥ 30 days) has been considered a worse prognostic factor [20], and intraoperatively placement of a TAFT [17] was reported to shortening the TPN duration to 12 days, in comparison to infants without feeding tube. In our Institution, placement of a TAFT has been routinely performed, as well as early attempt of weaning from TPN, resulting in a statistically significant difference among groups. The median duration of 10 days was present in groups A, B and D, while infants with VACTERL association had the median longer TPN duration (13 days) probably due to concomitant intestinal malformation. Earlier removing of central venous catheter and institutional infection surveillance protocols lowered the risk of sepsis, affecting only 8.9% in our sample.

### Length of stay

Prolonged LOS, also for corrected gestational age at discharge, was observed in infants belonging to groups C and D. These findings, related to the more complex global clinical profile of such infants, can be also ascribed for group C to the median longer duration of TPN, and for group D to the frequent central type hypotonia affecting feeding skills [22]. Early surgical complications (pneumothorax, chylothorax, anastomotic leakage, vocal cord paralysis) occurred in 15.6% of infants, without statistical difference among groups, thus not being considered responsible for prolonged LOS. Thus, we have attributed the observed higher LOS to longer period to achieve adequate alimentary autonomy to be discharged from hospital.

Our results are not completely in accordance with De Jong et al. [23] who reported a longer, but not statistically significant LOS in infants with non-isolated EA. We postulate that the different result in terms of statistical significance can be attributed to the more defined group subdivision we adopted. Thus, in our sample group B (EA with a single concomitant malformation) showed lower intensity level that the other “not isolated EA”.

## Conclusion

In our study we have found in infants with EA differences in intensive level care in accordance with the well-known classifications and related to individual auxological and pathological profile. Sample subdivision in four groups based on clinical presentation showed substantial differences, with the more complex conditions characterized by higher level of intensive care. Major cardiac anomaly was never found as unique malformation in not isolated EA. In infants with EA in the context of genetic and syndromic entities subjects with VACTERL association showed a lower mortality rate and a higher level of intensive care in comparison to infants with non VACTERL genetic and syndromic entities.

## Abbreviations

CHD: Congenital heart defects
EA: Esophageal atresia
IMV: Invasive mechanical ventilation
LGEA: Long gap EA
RDS: Respiratory distress
SGA: Small for gestational age
SGEA: Short gap EA
TAFT: Trans-anastomotic feeding tube
TBS: Tracheobronchoscopy
TEF: Tracheoesophageal fistula
